# Light Scattering by Vitreous of Humans With Vision Degrading Myodesopsia From Floaters

**DOI:** 10.1167/iovs.65.5.20

**Published:** 2024-05-10

**Authors:** Alba M. Paniagua-Diaz, Justin H. Nguyen, Pablo Artal, Wei Gui, J. Sebag

**Affiliations:** 1 Laboratorio de Óptica, Universidad de Murcia, Murcia, Spain; 2 VMR Institute for Vitreous Macula Retina, Huntington Beach, California, United States; 3 Doheny Eye Institute, University of California–Los Angeles, Pasadena, California, United States; 4 Department of Ophthalmology, Geffen School of Medicine, University of California–Los Angeles, Los Angeles, California, United States

**Keywords:** vitreous, vision degrading myodesopsia, light scattering, quantitative ultrasonography, contrast sensitivity, posterior vitreous detachment, myopic vitreopathy

## Abstract

**Purpose:**

Vision-degrading myodesopsia (VDM) from vitreous floaters significantly degrades vision and impacts visual quality of life (VQOL), but the relationship to light scattering is poorly understood. This study compared in vitro measures of light scatter and transmission in surgically excised human vitreous to preoperative indexes of vitreous structure, visual function, and VQOL.

**Methods:**

Pure vitreous collected during vitrectomy from 8 patients with VDM had wide-angle straylight measurements and dark-field imaging, performed within 36 hours of vitrectomy. Preoperative VQOL assessment with VFQ-25, contrast sensitivity (CS) measurements with Freiburg acuity contrast testing, and quantitative ultrasonography were compared to light scattering and transmission in vitro.

**Results:**

All indices of vitreous echodensity in vivo correlated positively with straylight at 0.5° (*R* = 0.708 to 0.775, *P* = 0.049 and 0.024, respectively). Straylight mean scatter index correlated with echodensity (*R* = 0.71, *P* = 0.04) and VQOL (*R* = −0.82, *P* = 0.0075). Dark-field measures in vitro correlated with degraded CS in vivo (*R* = −0.69, *P* = 0.04). VQOL correlated with straylight mean scatter index (*R* = −0.823, *P* = 0.012).

**Conclusions:**

Increased vitreous echodensity in vivo is associated with more straylight scattering in vitro, validating ultrasonography as a clinical surrogate for light scattering. Contrast sensitivity in vivo is more degraded in the presence of dark-field scattering in vitro and VQOL is decreased in patients whose vitreous has increased light scattering*.* These findings could form the basis for the development of optical corrections for VDM or support new laser treatments, as well as novel pharmacotherapy.

The vitreous body is 98% water, with hyaluronan and collagen constituting the major structural macromolecules.^1^^,^^2^ Light passing through clear vitreous experiences unhindered photon transmission to the retina. Opacities in the optical axis scatter light in all directions, with forward scattering (called *straylight*) interfering the most with vision. Measuring forward and backscattered light can provide insight into the impact of opacities on vision.

Fibrous liquefaction of vitreous occurs throughout life,^3^^–^^5^ culminating in posterior vitreous detachment (PVD) when there is concomitant dehiscence of vitreoretinal adherence.^6^^,^^7^ When fibrous liquefaction is advanced, such as in aging and myopia, patients experience the visual phenomenon of *floaters*.^8^ PVD exacerbates floaters, ostensibly because of the dense matrix of collagen in the posterior vitreous cortex and folds in the outer shell of the detached vitreous body.^9^ Severe cases merit the diagnosis of vision degrading myodesopsia (VDM), a term used to refer to clinically significant vitreous floaters based on objective measures of vitreous density and degradation in contrast sensitivity.^9^

Historically, vitreous floaters unrelated to retinal pathology have been dismissed as a “nuisance” and not considered a disease. Thus no treatments were offered to these patients who were simply advised to cope with their visual symptoms. Understandably, this has been perceived by most patients as an inadequate and frustrating recommendation. The underlying reason for this clinical approach is insufficient appreciation of the problem as a result of inadequate scientific information about the disease and the absence of ways to evaluate severity clinically. Visual acuity is usually unaffected, and the vitreous opacities that cause the visual phenomenon of floaters are often not visible to the examiner nor imaged by standard optical coherence tomography (OCT). To address the former limitation, contrast sensitivity (CS) has been determined to be a more sensitive measure of the visual impact of vitreous opacities than visual acuity.^8^^–^^10^ To address the latter limitation, improved imaging technologies with ultrasound scanning can enable more informative evaluation of vitreous structure in patients complaining of floaters.^11^^,^^12^ Although current OCT technology can image vitreous opacities that are close to the retina, these have never been quantified, and those in the central vitreous body cannot be reliably imaged by OCT. Thus quantitative ultrasonography (QUS) has been developed not only to image vitreous densities throughout the vitreous body but also to quantify their magnitude.^12^^,^^13^ These two clinical metrics (CS and QUS) enable the diagnosis of VDM.^9^^,^^10^

The mechanism(s) by which vitreous opacities and PVD cause VDM is/are presumed to be related to altered transmission of photons and untoward light scattering by the vitreous body, as well as the detached posterior vitreous cortex in patients with PVD.^8^^–^^10^ Although straylight has been measured,^14^ how contrast sensitivity and quantitative ultrasonography relate to altered photon transmission and light scattering has never been determined. This study therefore investigated the relationship between clinical evaluation of patients with VDM (by measuring CS, QUS, and VQOL) with objective measures of altered photon transmission and light scattering by vitreous of these same patients that was excised at vitrectomy surgery. It is hypothesized that there will be altered photon transmission, as well as abnormal light scattering, and that the degree of light scattering will correlate with the magnitude of objective measures of vitreous structure, visual function, and quality of life.

## Methods

This study adhered to the tenets of the Helsinki Agreement and obtained Institutional Review Board approval by Providence St. Joseph Health. Each subject elected vitrectomy as part of their routine clinical care and provided signed informed consent, obtained by co-author J.H.N. Visual function testing and structural imaging were performed prior to vitrectomy surgery. The study group (Table 1) consisted of eight subjects (four men and four women; 40–73 years old) with significant complaints of floaters of considerable duration (seven to 72 months, mean = 28.9 ± 24.9 months). All subjects had no prior history of vitreoretinal disease or therapy (surgery, injections, laser); in particular there was no personal or family history of inflammation or autoimmune disease. There were no cases of macular pucker membrane. Best-corrected visual acuity was 20/30 or better in all cases. Preoperative clinical characteristics are shown in Table 1.

**Table 1.** Study Group Clinical Characteristics

Patient I.D.	Age (Yrs)	Sex	Symptom Duration (Mo)	VA	Floater Etiology	Lens Status
1	73	F	48	20/30	PVD	PCIOL
2	51	F	10	20/25	PVD	Phakic
4	51	M	60	20/25	PPVD	Phakic
5	49	M	12	20/20	PPVD	Phakic
6	72	F	7	20/30	PVD	PCIOL
7	61	M	12	20/25	PVD	Phakic
8	65	F	72	20/20	PVD	PCIOL
9	40	M	10	20/20	PVD/MV	Phakic

MV, myopic vitreopathy; PCIOL, posterior chamber intraocular lens. PPVD, partial PVD; VA, visual acuity.

Each patient was evaluated by complete ophthalmologic examination (J.S.) and special testing (see below). PVD (confirmed by ultrasound scanning and OCT, see below) was the cause of symptoms in all subjects, and no subjects had vitreous opacities out of proportion to PVD. No subjects had evidence of pigmented cells in the vitreous body and 3/8 (37.5%) were pseudophakic, but these were monofocal intraocular lenses, which have been previously shown to only lower CS by 6.3% compared to phakic eyes.^15^ VQOL was assessed with the N.E.I. VFQ-25 that ranges from 0 to 100 (best).

### Visual Function

Visual function testing was performed in the same manner as previously described.^8^^,^^13^^,^^16^^–^^18^ Best-corrected visual acuity was recorded using a standard ETDRS backlit chart. Patients were then dark-adapted for 10 minutes before performing contrast sensitivity testing in a mesopic setting. A standard backlit LED computer monitor was employed to measure contrast sensitivity with the Freiburg acuity contrast test (FrACT).^19^^,^^20^ The software requires calibrations of screen resolution, fixed observer-screen testing distance, and a monitor luminance range of 80–320 cd/m² (DIN and ISO norms). Normal contrast sensitivity is typically better than conventional screen luminance resolution, which at 8 bits allows only 256 luminance levels. This is addressed by use of a technique known as *dithering* to improve luminance resolution.^21^ Dithering involves spatial pooling of each 2 × 2 block of pixels to achieve intensity values intermediate to those of single pixels. This allows subthreshold contrast stimuli to be generated, thereby increasing the effective resolution and transcending the 8-bit limitation of conventional displays. The Freiburg visual acuity contrast test uses psychometric function methodology where 18 trials with various levels of bright and dark luminance are displayed. The Weber index (%W) was selected to represent the results, because it has previously been established that this is preferred for letter stimuli, such as the Landolt C used in these studies.^21^ The Weber index is calculated as follows:

$$\begin{array}{ccc} & & \;\%\text{Weber}\;\left(\%W\right)=100\;\times \;\frac{Luminance_{max}-\;Luminance_{min}}{Luminance_{max}} \end{array}$$

The %W represents the contrast threshold (i.e., the contrast required to see the target reliably), whereas contrast sensitivity is the reciprocal of threshold.^22^ Hence, the higher the %W contrast threshold, the lower (worse) the contrast sensitivity.

### Vitreous Structure

Ultrasound scanning has been shown to be a useful noninvasive test to visualize abnormal tissue alterations related to changes in mass density (e.g., liquefied vs. gel vitreous). In this study, QUS was performed with AVISO (Quantel Medical, France), using a customized 15-MHz high-frequency single-element probe with a focused transducer of 20 mm focal length, 7 mm transducer aperture, and 6-dB bandwidth central frequency ranging from 9.5 to 20.0 MHz, as previously described.^11^^,^^12^ Ultrasonography was performed after visual function testing (see above). To maximize imaging quality, the transducer probe was placed directly on the globe after 0.5% proparacaine topical anesthesia was induced and topical 0.3% hypromellose served as a conductive medium. With the probe positioned nasal to limbus, 100 frames of imaging were recorded with the patient in temporal gaze so that a horizontal scan was achieved through the premacular central and posterior vitreous. Three QUS parameters were generated: sum of the square of the acoustic values within the region of interest (ROI) divided by the ROI area (energy), mean acoustic values divided by the ROI size (mean), and percentage of the ROI filled by clusters of echogenic regions greater in size than 50 pixels or 0.069 mm (P50). A QUS composite index has been used in previous studies^12^^,^^13^ and is defined as follows:

$$\begin{array}{ccc} & & \;\text{Composite}\;\text{QUS}\;\text{Index}\\ & & \;=\;\frac{1}{2}\;Energy+\left(10\;\times Mean\right)+\left(100\times P50\right). \end{array}$$

### Vitreous Sample Procurement

Vitrectomy was performed (W.G. & J.S.) with 25-gauge instruments (Constellation; Alcon, Ft. Worth, TX, USA) at a cutting rate of 2500 cpm. At the very beginning of surgery and before any manipulation of the vitreous body, approximately 1.0 mL of pure undiluted vitreous was obtained via automated cutting at a constant rate of 2500 cpm with manual aspiration using a 1 mL syringe attached to the stopcock in the vitrectomy aspiration line. The fresh, undiluted, unfixed vitreous samples were stored at room temperature, and light scattering measurements were performed individually in each sample within 36 hours of acquisition. There were no postoperative complications such as infection, vitreous hemorrhage, or retinal tears/detachments.

### Light Scattering Measurements

Light originating from an external object travels through a clear eye in straight lines that can be described by geometrical optics which characterize unhindered photon transmission. However, when photons encounter scattering objects (opacities), light is scattered in all directions, causing visual impairment in the form of blurring, reduced contrast, or shadows. The major nuisance(s) arise(s) from forward scattered light that reaches the retina, which is called “straylight.” The optical integration method^23^ has been demonstrated to be an excellent approach to quantify forward scattering straylight. Scattering can be also quantified by measuring the reduction in transmitted light intensity, using the well-known Lambert-Beer Law. However, because of the sparsity of vitreous opacities, it was very challenging with available power meters to measure the change in transmission; therefore we used a sensitive camera for that purpose and quantified the scattered light using the mean scatter index, as we describe below. Furthermore, because backward scattered light is proportional to the forward scattered light, we also measured this by taking dark field images of the samples, quantifying it also by using the mean scatter index, as described below.

#### Objective Straylight Characterization

When light enters the eye en route to the retina, it traverses through different media. The term *straylight* refers to intraocular scattered light. The angular dependence of the straylight (*s*) is given by the product between the point spread function (PSF) and the angle (θ) squared:

(1) $$\begin{array}{c} s\left(\theta \right)=\theta^{2}PSF\left(\theta \right) \end{array}$$

The PSF of the samples as a function of θ was measured using the optical integration method.^23^^,^^24^ This technique is based on the projection of monochromatic-light disks of different diameters (2θ) and uniform irradiance through the vitreous samples, whereas the central intensity (I_c_(θ)) of the images was measured. I_c_ is the summation of the weighted contributions PSF(φ) from all the bright sources at a radial distance φ within the disk:

(2) $$\begin{array}{c} I_{c}\left(\theta \right)=\int_{0}^{\theta }2\pi \varphi PSF\left(\varphi \right)d\varphi \end{array}$$

Once I_c_(θ) is normalized to its maximum value, PSF(θ) is given by the following:

(3) $$\begin{array}{c} PSF\left(\theta \right)=\frac{1}{2\pi \theta }\frac{dI_{c}\left(\theta \right)}{d\theta } \end{array}$$

**Figure 1.**
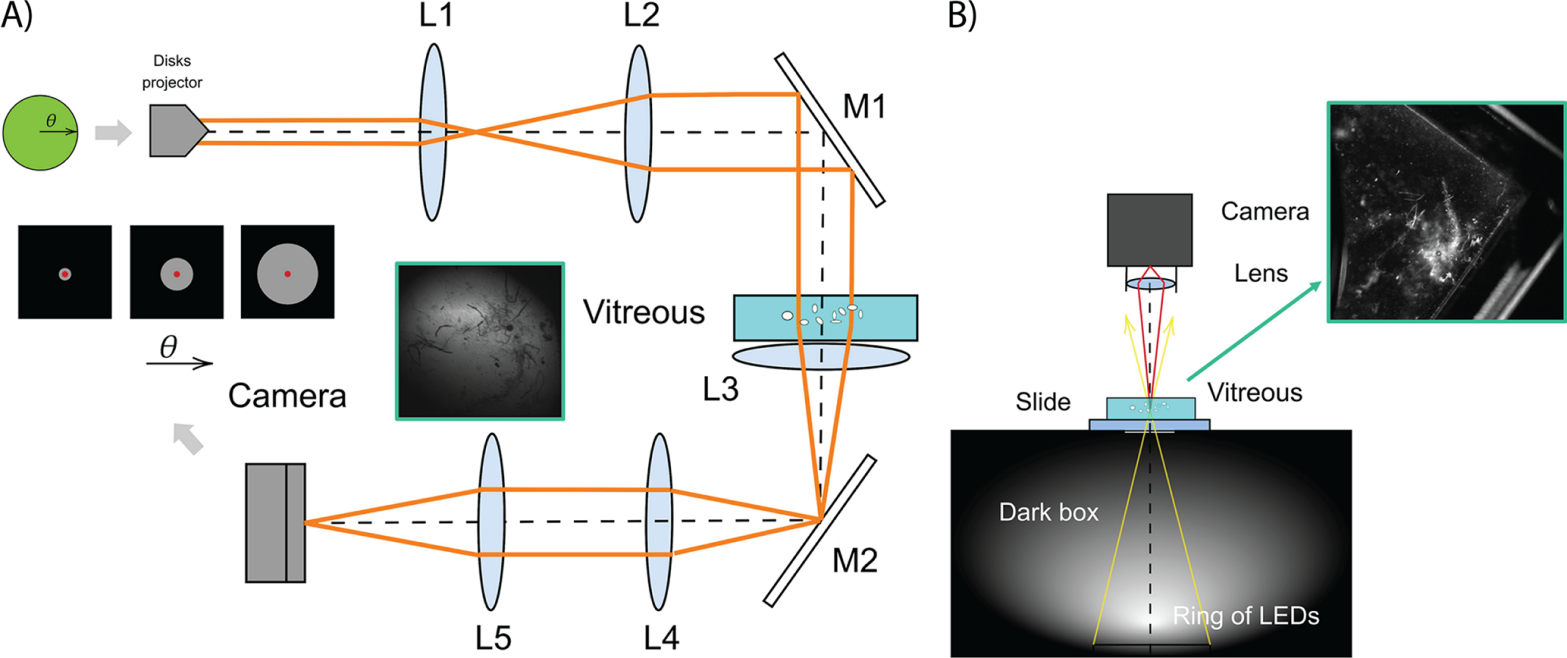
(**A**) Schematics of the optical integration method used and image of the back-illuminated sample (**inset**). (**B**) Dark field imaging technique and an example of a sample's image in the inset. L= lens, M = mirror, *Ɵ* = angle.

Figure 1A shows the experimental setup of the optical integration method. The disks were displayed with a full high-definition liquid crystal on silicon screen (Syndiant 2281; Syndiant Inc., Dallas, TX, USA) illuminated with a Xenon lamp (Model E7536; Hamamatsu Photonics, Hamamatsu, Japan). A telescope composed of lenses L1 and L2 conjugates the entrance pupil with the sample plane, which is immersed in a cuvette with Eusol-c. The illumination diameter on the sample was 5 mm. A spectral filter (FB550-40; Thorlabs, Newton, NJ, USA) was placed in front of L1 to produce a quasi-monochromatic illumination with central wavelength 550 ± 8 nm.

Samples of pure, undiluted vitreous were placed into glass cuvettes of dimensions 20 × 5 × 40 mm. A 100 mm lens (L3) was placed adjacent to the cuvette to aid focusing and form the PSF on the imaging camera. For each sample, the disks were imaged using a complementary metal-oxide-semiconductor (CMOS) camera (DCC1545M; Thorlabs) and a final telescope formed by L4 and L5 lenses. The amount of baseline straylight within the optical system was determined when the cuvette was filled with water. For the straylight calculations, several disks of diameter 2θ were displayed, and the central intensity of their images (the position marked by the red dot in Fig. 1A) was recorded. The angular distribution of the PSF was then calculated from the angular local slopes of the central intensities, according to Equation 3. For each straylight measurement, 114 disks were projected, where the processed central intensity corresponds to an average of three sequential measurements.

The maximum angular range of the measured PSF was 6.1°. This value was determined by the size of the display projecting the disks (0.5-inch display) and the focal length of the collimating lens (35 mm focal length). The PSFs retrieved using Equation 3 were normalized on the solid angle of 25.9 × 10^−3^ stereo-radian (sr). The units of the PSF and *s* are sr^−1^ and deg^2^sr^−1^, respectively, although the logarithmic values of the straylight (Log_10_(s)) are used to compare with other approaches.^14^ Because of the dilute nature of the sample, we used the values at 0.5° from the whole angular range as reference for straylight intensity, accurately accounting for the decay of the PSF peak intensity as a consequence of the low scattering properties of the sample. The dark noise of the camera and the effects of parasitic light were also taken into account by subtracting the intensity (at the center of the disks) when no image was displayed on the projector, for the same camera settings.

Using this same setup, we adjusted the axial position of the camera to image the sample plane, as shown in the blue inset of Figure 1A. Using the same integration time of the camera in all measurements we measured the straylight mean scatter index (straylight MSI). As a relative measurement, this provides comparative information regarding the transmitted intensity (the one that is not occluded by vitreous opacities). This value is obtained by choosing five different regions of the image around the opacities and performing the normalized average intensity in the chosen region. Because the CMOS camera used is an 8-bit camera, the values ranged between 0 and 255. Given that the measurements are taken in transmission, the vitreous opacities appear as black, impeding light from reaching the camera, so the more (larger in size or greater in number) opacities present in a sample, the smaller the transmitted intensity and consequently the greater the straylight MSI.

#### Dark-Field Imaging

Dark-field (DF) images of these vitreous samples were also recorded to further evaluate human vitreous in vitro. As described above, a comparative quantification of the scattering was performed by determining the mean scatter index (MSI), quantifying the average reflected light intensity taken in five different locations including the opacities. Figure 1B shows the schematic of the custom-built system for the acquisition of dark-field images. This system is a black box with a small square aperture at the top (10 × 10 mm). Inside the black box, at the bottom there is a ring of white LEDs, whose light passes through the central hole where the vitreous sample is placed. Because of the off-axis illumination, light that reaches the camera is imaged by a CMOS camera (UI-324xCP-NIR; IDS Imaging Development Systems GmbH, Obersulm, Germany). The parameters of the camera (i.e., gain and exposure time), as well as the illumination power of the LEDs were fixed for the acquisition of all tested vitreous, allowing comparisons among them. The inset in Figure 1B shows an example of a vitreous sample in the measuring cuvette, where the whitish areas correspond to the ones generating more scattering, because of the reflective nature. This camera is also an 8-bit camera, with values ranging from 0 to 255; here, larger values of intensity are opposite from the straylight MSI case. To account for inhomogeneity of the samples, five measurements were taken in different regions of the sample for the quantification of the MSI both for the transmissive (straylight) and reflective (dark-field) imaging configuration, enabling an adequate averaging of the global scattering of the vitreous sample itself.

### Statistical Analyses

Clinical indexes and light scatter indexes were recorded for each of the eight subjects. Basic statistics of mean, standard, deviation, and range were calculated for all indexes (CS, QUS, and Light Scatter). A scatterplot was generated to display the relationship between clinical indices and light scattering parameters. A Pearson correlation coefficient was calculated for each comparison.

## Results

Table 2 presents all in vivo clinical test findings. Contrast threshold ranged from 3.03 to 5.31 %W, averaging 4.3 ± 1.3 %W (higher value is worse contrast sensitivity), which is consistent with our previously published data where control subjects had an average of 2.4 %W, while patients complaining of significant floaters (i.e., elected vitrectomy) had an average of 4.6 %W.^13^

**Table 2.** Preoperative and Postoperative Clinical Test Results

Subject ID	VFQ-25 (77.8)	CS (%W)	QUS-Mean (AU)	QUS-Energy (AU)	QUS-P50 (AU)	QUS-Composite (AU)
1	78.3	6.26	29.7	1098.2	7.8	1626.2
2	84.7	2.34	21.7	514.8	0.1	489.1
4	75.0	4.17	26.1	852.6	5.1	1192.3
5	63	3.03	26.8	887.2	6.1	1317.4
6	71	5.15	26.5	1019.6	8.5	1621.1
7	77	5.25	31.9	1359.5	8.7	1931.1
8	71	3.62	28.5	1023.9	7.0	1492.6
9	77.8	4.54	29.9	1060.7	6.3	1457.9
Preoperative Mean ± SD	74.7 ± 6.5	4.3 ± 1.3	27.6 ± 3.1	977.1 ± 241.8	6.2 ± 2.7	1391 ± 426.5
Postoperative Mean ± SD	85.8 ± 6.8	2.2 ± 0.4	22.9 ± 1.9	606 ± 133	1.2 ± 1.5	654.4 ± 231
*P*	0.0049	0.0006	0.0026	0.0019	0.0004	0.0007

QUS-Mean, mean acoustic values divided by the ROI size; QUS-Energy, sum of the square of the acoustic values within the ROI divided by the ROI area; QUS-P50, percentage of the ROI filled by clusters of echogenic regions greater in size than 50 pixels or 0.069 mm; QUS-Comp, composite index of QUS measurements (see Methods).

All these values are consistent with previous studies of patients with vision degrading myodesopsia from vitreous floaters.^11^^,^^13^^,^^17^^–^^19^^,^^23^

QUS determined the following: QUS-Mean = 27.6 ± 3.1 (arbitrary units [AU]), QUS-Energy = 977.1 ± 241.8 (AU), QUS-P50 = 6.2 ± 2.7 (AU), and QUS-Composite = 1391 ± 426.5 (AU); all similar to previous findings in patients with VDM.^9^^,^^11^^–^^13^ After surgery visual acuity was unchanged, CS improved by 49% (*P* = 0.0006), vitreous echodensity decreased in all indexes (Table 2) with the QUS composite decreasing by 53% (*P* = 0.0007), and VQOL (VFQ-25) improved by 13%.

Results of in vitro light scattering measurements in excised human vitreous are presented in Table 3. Straylight at 0.5° averaged 1.45 ± 0.29, straylight MSI = 0.21 ± 0.07, and dark-field MSI = 36.2 ± 10.7. These were all distinctly different from controls of clear water.

**Table 3.** Light Scattering by Human Vitreous

Subject	Str @ 0.5°	Str MSI	DF MSI
1	1.3	0.16	33.2
2	0.96	0.14	23.4
4	1.32	0.11	21.6
5	1.64	0.2	35.2
6	1.36	0.28	44
7	1.88	0.32	54.4
8	1.72	0.24	36.4
9	1.42	0.24	41.2
Mean ± SD	1.5 ± 0.3	0.2 ± 0.1	36.2 ± 10.7

Str @ 0.5°, straylight at 0.5°; Str MSI, straylight MSI.

**Figure 2.**
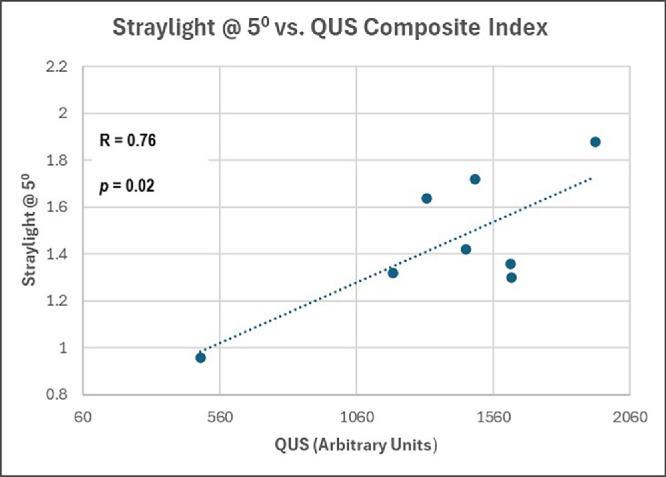
Straylight correlation with quantitative ultrasonography. In vitro straylight at 0.5° (y-axis) increased with increasing in vivo vitreous echodensity (x-axis), as measured with the composite index of all quantitative ultrasonography parameters (see methods) (*R* = 0.76, *P* = 0.02).

**Figure 3.**
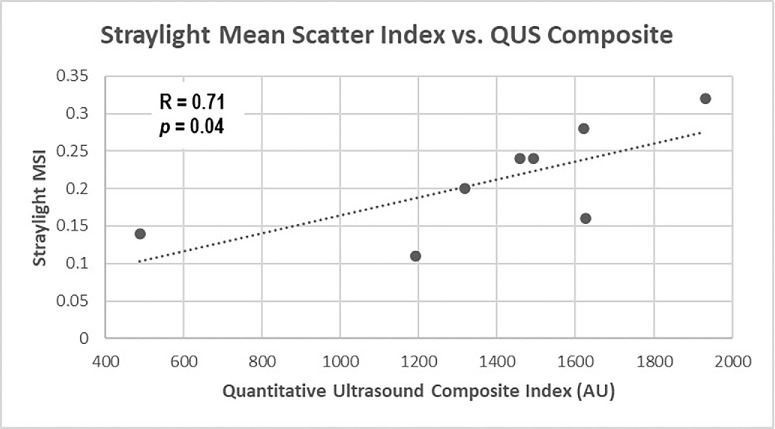
Straylight MSI correlation with QUS. With increasing vitreous echodensity (QUS composite index [see Methods] on x-axis) in vivo, there was increased straylight scattering in vitro (y-axis) (*R* = 0.71, *P* = 0.0388).

**Figure 4.**
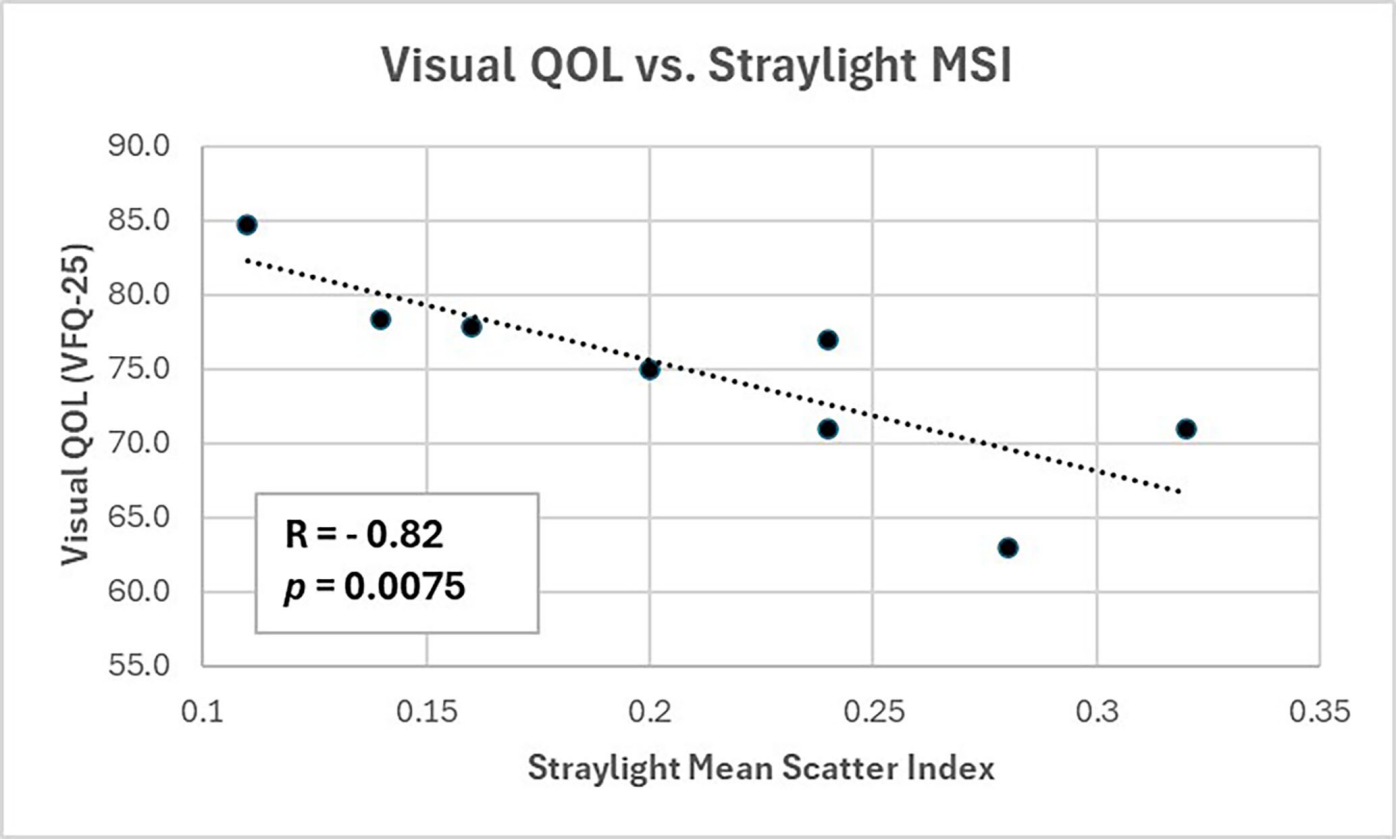
Straylight MSI correlation with visual quality-of-life. With increasing straylight (Straylight MSI, x-axis) in vitro, there was decreasing visual quality of life in vivo (y-axis, VFQ-25) in vivo (*R* = −0.82, *P =* 0.0075).

**Figure 5.**
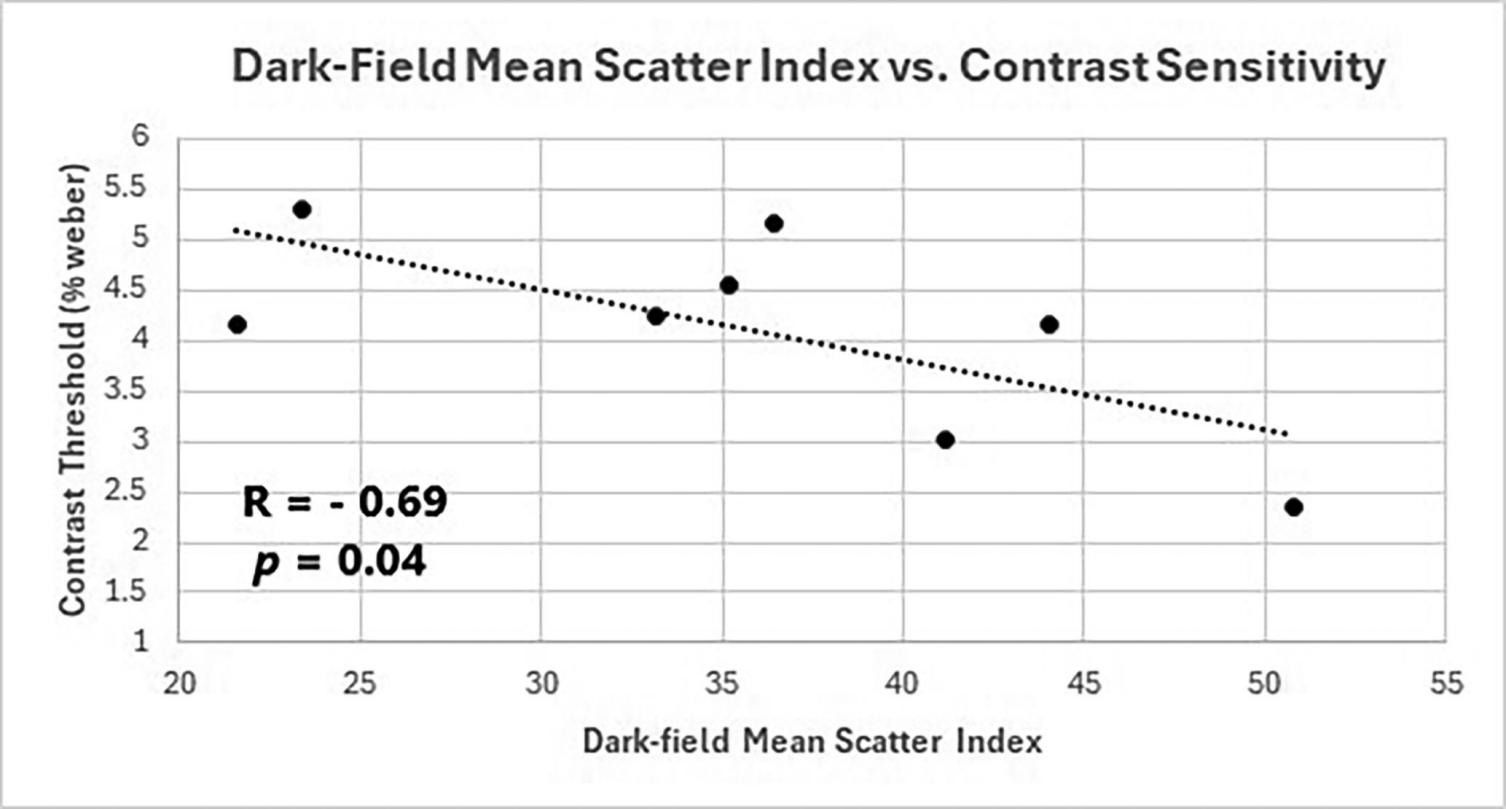
Contrast sensitivity correlation with dark-field MSI. With increasing back scattering (dark-field MSI, on x-axis) in vitro, there was worse contrast sensitivity in vivo (higher contrast threshold (% Weber on y-axis) (*R* = −0.6887, *P* = 0.0484), most likely because of diminution in transmission of photons by vitreous opacities. Thus the findings are opposite of those observed with straylight measurements, as predicted above (see Methods).

The three light scattering indexes were correlated with the three clinical indexes (VQOL measured by VFQ-25; vision, measured by CS; and vitreous echodensity, measured by QUS). All correlations showed positive trends, with four correlations attaining statistical significance: straylight at 0.5° correlated with the composite index of QUS (*R* = 0.76, *P* = 0.0206; Fig. 2), straylight MSI correlated with the composite index of QUS (*R* = 0.71, *P* = 0.0388; Fig. 3), and with VFQ-25 (*R* = −0.82, *P* *=* 0.0075; Fig. 4), whereas dark-field MSI correlated with contrast sensitivity (CS; *R* = −0.69, *P* = 0.0484; Fig. 5). As previously stated, larger values of DF intensity are opposite from the Straylight MSI; hence, higher DF MSI values corresponded to lower %W, which is opposite of what was observed with QUS and straylight measurements.

VQOL correlated strongly (*R* = −0.823, *P* = 0.012) with light scattering, so that the more the measured straylight MSI (x-axis), the worse the VQOL as evaluated with VFQ-25 questionnaire (y-axis). Once again, this resulted in a negative R-value (see Fig. 3).

As indicated in Table 4, there were strong positive correlations between straylight (Str @ 0.5°) and each QUS parameter: QUS-Energy (*R* = 0.755, *P* = 0.030), QUS-Mean (*R* = 0.775, *P* = 0.024), QUS-P50 (*R* = 0.708, *P* = 0.049), as well as the composite index of all three parameters: QUS-Composite (*R* = 0.761, *P* = 0.028). The MSI of straylight (Str MSI) revealed strong positive correlations between QUS-Energy (*R* = 0.727, *P* = 0.041) and QUS-Composite (*R* = 0.710, *P* = 0.048) and trended toward statistical significance correlation for QUS-Mean (*R* = 0.610, *P* = 0.108) and QUS-P50 (*R* = 0.665, *P* = 0.072).

**Table 4.** Correlations Between Quantitative Ultrasonography In Vivo and Vitreous Light Scattering In Vitro

	Str @ 0.5°		Str MSI	
	*R*	*P*	*R*	*P*
QUS-Energy	0.775	0.024^*^	0.727	0.041^*^
QUS-Mean	0.755	0.030^*^	0.610	0.108
QUS-P50	0.708	0.049^*^	0.665	0.072
QUS-Composite	0.761	0.028^*^	0.710	0.048^*^

QUS-Energy, sum of the square of the acoustic values within the ROI divided by the ROI area; QUS-Mean, mean acoustic values divided by the ROI size; P50, percentage of the ROI filled by clusters of echogenic regions greater in size than 50 pixels or 0.069 mm; QUS-Composite, composite index of QUS measurements (see Methods); Str @ 0.5°, straylight at 0.5°; Str MSI, Straylight MSI.

* High correlation coefficients (*R* > 0.7) with statistical significance (*P* < 0.05) between all QUS parameters with Str @ 0.5°, between QUS-Energy (*R* = 0.727, *P* = 0.041) with Str MSI, and between QUS-Composite with Str MSI. There were strong trends toward statistical significance between QUS-Mean (*R* = 0.610, *P* = 0.108) and QUS-P50 (*R* = 0.665, *P* = 0.072) with Str MSI. There were no correlations between ultrasonography and dark-field mean scatter index (*R* ranged from −0.240 to −0.398, *P* ranged from 0.319 to 0.546).

## Discussion

VDM results from disturbances induced by vitreous opacities that create images described by patients as *floaters*. Although this condition has historically been considered by doctors as a nuisance and not a disease, these visual obscurations apparently have a significant impact on VQOL.^25^^,^^26^ It has long been presumed that the visual symptoms of floaters result from untoward light scattering by vitreous opacities, but data supporting this relationship have been lacking. The present study determined that in vitro light scattering and altered photon transmission by surgically excised vitreous samples from patients complaining of floaters and who are afflicted with VDM is detectable and correlates with in vivo preoperative clinical metrics of vitreous echodensity, visual function, and visual quality of life.

The significantly positive correlations between ultrasound measures of vitreous density and light scattering (straylight at 0.5° and Straylight MSI) validate the use of ultrasonography as a reasonable clinical surrogate for measuring light scatter by vitreous in vivo. This likely explains the past utility with quantitative ultrasonography as a reliable measure of vitreous density in myopia,^27^ aging,^19^ and after posterior vitreous detachment.^18^ Quantitative ultrasonography was also previously found to correlate with the negative impact on VQOL^11^ and was recently determined to be machine-independent.^12^ Perhaps most importantly, quantitative ultrasonography effectively assessed the salubrious response to surgery with vitrectomy,^13^^,^^17^ as well as the variable effects of YAG laser vitreolysis.^28^ The relationship between VQOL, quantitative ultrasonography, and light scattering is corroborated by the results of the present study, substantiating the value of quantitative ultrasonography in clinical practice. However, the utility of ultrasonography notwithstanding, there is great potential diagnostic and therapeutic significance to measuring light scatter by vitreous in both health and disease^29^; hence, future studies should use existing technologies and develop new methods with which to quantify vitreous light scattering in patients.

There are some limitations to this study. The most significant is the small sample size of the study population. This likely influenced the weak correlations between straylight MSI and the QUS-P50, as well as the QUS-Mean indexes of vitreous echodensity, which trended toward but did not attain statistical significance (*R* = 0.665, *P* = 0.072 and *R* = 0.61, *P* = 0.108; respectively). Furthermore, other correlations that trended but did not attain statistical significance (see above) were also likely limited by the small study population. However, it should be noted that the light scattering measurements had a definite distinction from the benchmark reference of a water-filled cuvettes and were accurate enough to characterize light scattering in excised vitreous samples which correlated strongly with vitreous structure and visual function in several comparisons. Furthermore, this approach was used in a recent study of enzyme effects on vitreous in vitro*,* substantiating the value of measuring forward light scattering by vitreous in vitro,^30^ as was performed in the present study. Another challenge in conducting this research, however, was the sparsity of opacities in the vitreous samples, which posed difficulties in accurately measuring a wide-angle straylight parameter. Because measurements exhibited variability and fluctuations, particularly for angles exceeding 0.5 degrees, these were deemed less dependable, and as a result, we relied exclusively on measurements up to this angular threshold for the in vitro analyses. Last, vitreous samples were obtained using standard vitrectomy instrumentation that mechanically cuts vitreous, possibly introducing artefact.

In conclusion, measures of photon transmission and light scattering by vitreous obtained from patients with VDM correlate with QUS, CS, and VQOL. This validates the use of ultrasonography as a clinical surrogate for light scattering. More importantly, however, these results define the association between altered photon transmission (dark-field measures), light scattering (straylight), and contrast sensitivity degradation, as well as reduced VQOL. Thus, further investigations into the impact(s) of abnormal vitreous on photon transmission and untoward light scattering should be undertaken, because this can potentially lead to the development of optical correction techniques for Vision Degrading Myodesopsia, as has been previously proposed.^31^ Additionally, such studies might pave the way for innovative laser treatments or new pharmacotherapies tailored to address vitreous abnormalities.^32^^,^^33^
